# Neuropathological features of levodopa-responsive parkinsonism in multiple system atrophy: an autopsy case report and comparative neuropathological study

**DOI:** 10.3389/fneur.2023.1293732

**Published:** 2023-11-14

**Authors:** Mitsuyoshi Tamura, Takahiro Takeda, Yoshihisa Kitayama, Tomoki Suichi, Kazumoto Shibuya, Sakurako Harada-Kagitani, Takashi Kishimoto, Satoshi Kuwabara, Shigeki Hirano

**Affiliations:** ^1^Department of Neurology, Graduate School of Medicine, Chiba University, Chiba, Japan; ^2^Department of Neurology, National Hospital Organization Chiba Higashi Hospital, Chiba, Japan; ^3^Department of Molecular Pathology, Graduate School of Medicine, Chiba University, Chiba, Japan

**Keywords:** multiple system atrophy, levodopa, putamen, pathology, presynaptic dopaminergic fibers

## Abstract

**Background:**

In typical patients with multiple system atrophy with predominant parkinsonism (MSA-P) levodopa is ineffective. However, there are some of these patients who respond well to levodopa treatment. Levodopa efficacy in MSA-P patients is thought to be related to the degree of putaminal damage, but the pathological causation between the putaminal involvement and levodopa efficacy has not been established in detail.

**Objective:**

This study aimed to evaluate the neuropathological features of the nigrostriatal dopaminergic system in a “levodopa-responsive” MSA-P patient in comparison with “levodopa-unresponsive” conventional MSA-P patients.

**Materials and methods:**

Clinicopathological findings were assessed in a 53-year-old Japanese man with MSA who presented with asymmetric parkinsonism, levodopa response, and later wearing-off phenomenon. During autopsy, the nigrostriatal pathology of presynaptic and postsynaptic dopaminergic receptor density and α-synuclein status were investigated. The other two patients with MSA-P were examined using the same pathological protocol.

**Results:**

Four years after the onset, the patient died of sudden cardiopulmonary arrest. On autopsy, numerous α-synuclein-positive glial cytoplasmic inclusions in the basal ganglia, pons, and cerebellum were identified. The number of neurons in the putamen and immunoreactivity for dopamine receptors were well-preserved. In contrast, significant neuronal loss and decreased dopamine receptor immunoreactivity in the putamen were observed in the “levodopa-unresponsive” MSA-P control patients. These putaminal pathology results were consistent with the findings of premortem magnetic resonance imaging (MRI). All three patients similarly exhibited severe neuronal loss in the substantia nigra and decreased immunoreactivity for dopamine transporter.

**Conclusion:**

Levodopa responsiveness in patients with MSA-P may be corroborated by the normal putamen on MRI and the preserved postsynaptic nigrostriatal dopaminergic system on pathological examination. The results presented in this study may provide a rationale for continuation of levodopa treatment in patients diagnosed with MSA-P.

## Introduction

Multiple system atrophy (MSA) is a sporadic neurodegenerative disease that presents with autonomic dysfunction, parkinsonism, and cerebellar ataxia. The typical phenotypes of MSA are MSA with predominant parkinsonism (MSA-P) and MSA with predominant cerebellar ataxia (MSA-C). The pathological hallmark of MSA is the presence of α-synuclein-positive glial cytoplasmic inclusions (GCIs) in the oligodendrocytes and neuronal cytoplasmic inclusions (NCIs) across the central nervous system (1, 2). However, owing to the variable neurological manifestations and clinical courses of MSA, especially in the early stages of the disease, it is sometimes difficult to differentiate it from other neurological conditions, such as Parkinson’s disease (PD) or progressive supranuclear palsy (PSP) (3).

Parkinsonism in patients with MSA is typically characterized by a poor and unsustained response to levodopa treatment (4, 5), although a previous study found that 12–68 (17.6%) patients with MSA demonstrated levodopa-responsive parkinsonism (6). It has been hypothesized that the response to levodopa depends on putaminal involvement (7). However, this has not yet been confirmed by clinicopathological analyses. Pathological and neuroimaging comparisons of putaminal lesions between MSA levodopa responders and non-responders have been inconclusive.

Recently, we encountered a patient who presented with a 4-year history of parkinsonism, which responded well to levodopa in addition to his end-of-dose off phenomenon and was pathologically diagnosed with MSA. This study aimed to describe the clinical, neuroimaging, and neuropathological characteristics of this patient. Furthermore, this study aimed to evaluate the pathological differences between this patient and other neuropathologically confirmed patients with MSA-P in the context of levodopa responsiveness. Notably, to address the pathological features of levodopa responsiveness, pre- and postsynaptic degenerations in the nigrostriatal system were investigated.

## Materials and methods

### Participants

Including the current patient in this study, three patients with MSA-P (one man and two women, pathologically diagnosed from 2010 to 2022) were enrolled in the neuropathological analyses (Table 1). Informed consent was obtained from the patients or their families for clinical and autopsy analyses. All procedures were performed in accordance with the ethical standards of the institutional research committees and the 1964 Declaration of Helsinki and its later amendments or comparable ethical standards. This study was approved by the Institutional Review Board of Chiba University (#3469). The presentation of the current patient showing a good response to levodopa treatment is described in the Results section.

**Table 1:** Demographics and neuropathological feature of three cases with MSA-P.

Case	1 (present case)	2	3
MSA subtype	MSA-P	MSA-P	MSA-P
Age at death (years)	57	67	75
Sex	M	F	F
Disease duration (years)	4	5	5
Putaminal atrophy on MRI	−	+	+
Levodopa response	Good	Poor	Poor
Brain weight (g)	1,350	1,140	1,200
Lewy pathology	Absent	Absent	Present
Braak NFT staging	1	1	1

MSA, multiple system atrophy; NFT, neurofibrillary tangle.

### Clinical evaluation

In the current patient, all consultations and assessments during hospitalization were carried out by the same neurologist (M.T.). Clinical information on the control patients was obtained retrospectively from their medical records.

### Neuroimaging

T2 weighted imagings on magnetic resonance (MR) study were obtained in the current patient and the control patients. In the current patient, ^123^I-iodoamphetamine (^123^I-IMP) single-photon emission computed tomography (SPECT) was examined at the initial visit and 4 years after onset. Metaiodobenzylguanidine (^123^I-MIBG) myocardial scintigraphy and dopamine transporter (DAT) scan were examined at the initial visit. Each imaging conditions are available in the Supplementary material.

### Neuropathology

Brain and spinal cord tissues, including those of the midbrain and basal ganglia, were fixed in formalin for at least 2 weeks. Subsequently, 5-μm-thick sections of paraffin-embedded tissue were stained with hematoxylin and eosin, and Gallyas silver staining was performed in the representative sections. The sections were also immunostained for phosphorylated α-synuclein (pSyn#64, 1:10,000; FUJIFILM Wako Pure Chemical Corp., Osaka, Japan), phosphorylated tau (monoclonal, AT8, 1:500; CosmoBio, Tokyo, Japan), DAT (polyclonal, SLC6A3, 1:600; Bioss Antibodies Inc., Woburn, MA), dopamine receptor D1 (DRD1) (polyclonal, 17934-1-AP, 1:400; Proteintech Group, Inc., Rosemont, IL), and dopamine receptor D2 (DRD2) (polyclonal, 55084-1-AP, 1:600; Proteintech Group, Inc., Rosemont, IL). For α-synuclein, formic acid pretreatment (for 5 min) was performed. The biotinylated secondary antibody was detected using the streptavidin-biotinylated peroxidase complex method coupled with diaminobenzidine (DAB) reaction. Pathological statuses, such as brain weight, presence or absence of Lewy pathology, and Braak’s staging of neurofibrillary tangles (NFTs), were assessed (8).

Sections of the midbrain (including the substantia nigra [SN]) and basal ganglia (including the dorsolateral part of the putamen) were prepared to comparatively investigate the severity of nigrostriatal degeneration, its relationship with neuronal loss between the SN and putamen, putaminal immunoreactivity of DAT (as a presynaptic marker of the nigrostriatal system) and DRD1 and DRD2 (as postsynaptic markers of the nigrostriatal system), and α-synuclein pathology in patients with MSA. The sections stained with hematoxylin and eosin were used to calculate the neuron packing density. Ten fields of view (view size: 0.1243 mm^2^) at 400-fold magnification were randomly selected in each region, and the melanin-containing neurons in the SN and small- and large-sized neurons in the putamen were counted. Cells were counted using a light microscope (Olympus BX53 + DP74; Olympus, Tokyo, Japan). Cells mimicking neurons and glia were excluded from counting. To calculate the DAT-positive, DRD1-positive, DRD2-positive, and α-synuclein-positive areas intensified with brown color by DAB chromogen, a binarization method was applied with a threshold value of 95. This threshold value was set in advance as follows: pixel brightness (with a range of 0–256) was calculated in randomly selected 100 DAT-, DRD1-, DRD2-, and α-synuclein-positive (DAB-positive) structures and 100 negative (DAB-negative) structures. Subsequently, two brightness graphs were plotted, and the brightness threshold was obtained from the overlapping portions of the two graphs. The mean immunoreactive areas (DAT-, DRD1-, DRD2-, and α-synuclein-positive %area) were calculated by dividing the mean DAT-/DRD1-/DRD2−/α-synuclein-positive number of pixels by the total number of pixels in the entire images (Photoshop CC, Adobe Inc., San Jose, CA, United States).

## Results

### Case presentation

**Figure 1:**
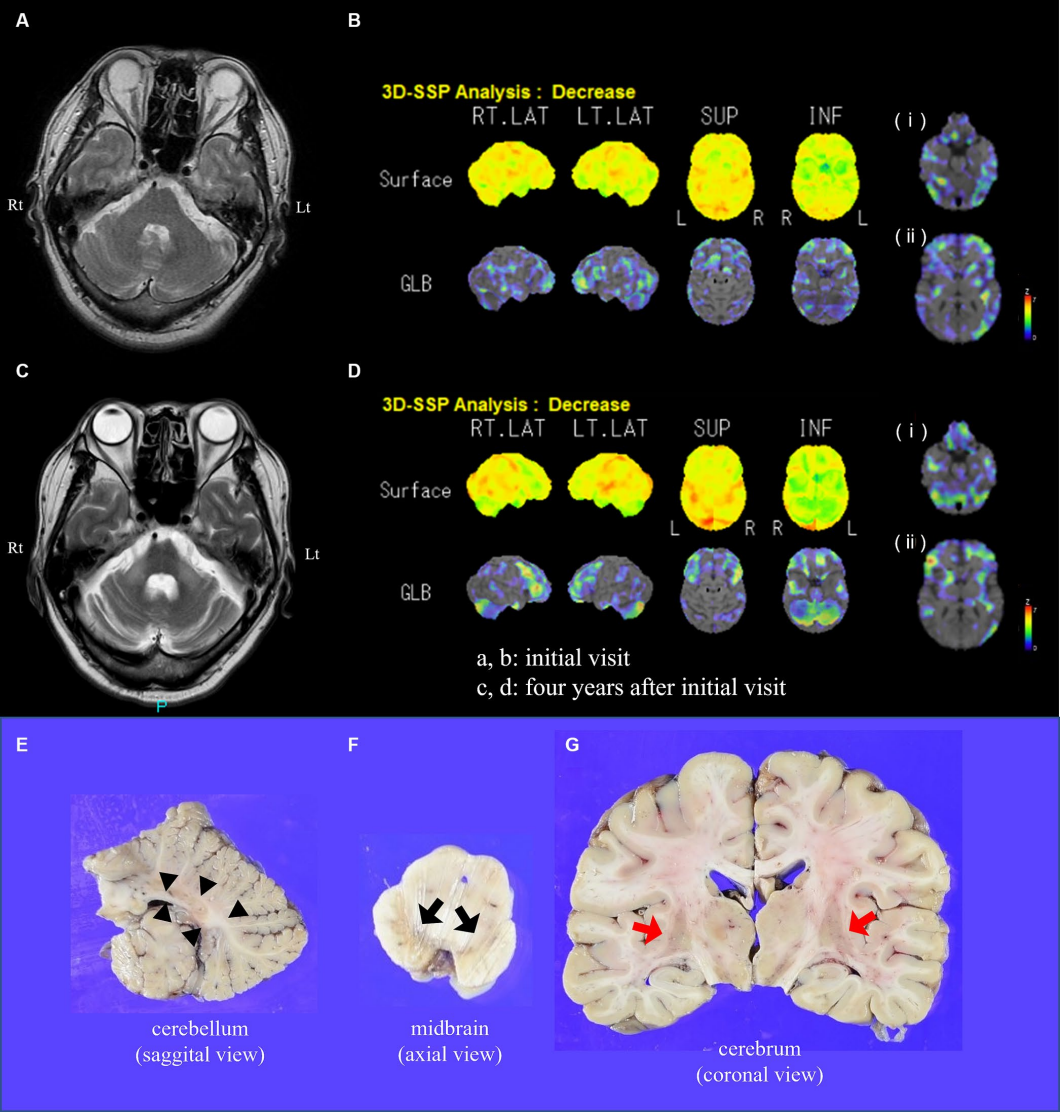
Brain MRI, perfusion imaging, and macroscopic appearance of the brain in the present case. At initial visit, **(A)** T2-weighted imaging on brain MRI and **(B)** three-dimensional stereotactic surface projection (3D-SSP) analyses on ^123^I-IMP SPECT at the level of (i) the brainstem and cerebellum and (ii) striatum reveal no evident abnormalities. Four years after initial visit, **(C)** T2-weighted imaging reveals marked cerebellar atrophy, hot cross bun sign, and high intensity of middle cerebellar peduncles. **(D)** 3D-SSP analyses on ^123^I-IMP SPECT reveal (i) decreased blood flow in the bilateral cerebellum and (ii) mild decreased blood flow in the right striatum. The sectional views of the cerebellum **(E)**, midbrain **(F)**, and cerebrum **(G)** are shown. **(E)** Modest atrophy, notably in the white matter (arrowheads), of the cerebellum is shown. **(F)** Severe depigmentation of the substantia nigra is evident (black arrows). **(G)** The putamen shows slightly brownish color and mild atrophy (red arrows). MRI, magnetic resonance imaging; ^123^I-iodoamphetamine single-photon emission computed tomography, ^123^I-IMP SPECT; L(t), left side; R(t), right side.

A 53-year-old Japanese man presented with a 4-month history of difficulty in moving his left hand and a 2-month history of left limb resting tremors and speech difficulties. During the first visit, left-dominant parkinsonism and a resting tremor were observed. He also had a medical history of hypertension, hyperuricemia, uveitis, appendicitis, and childhood asthma, but he had no history of heavy alcohol intake. His son had been diagnosed with intellectual disability and epilepsy. Brain magnetic resonance imaging (MRI) and ^123^I-IMP SPECT imaging revealed no evident abnormalities (Figures 1A,B). ^123^I-MIBG myocardial scintigraphy was normal in both the early and delayed phases. DAT imaging revealed a right-dominant decrement, indicating presynaptic dopaminergic dysfunction. His motor symptoms were improved after treatment with 300 mg/day levodopa/benserazide, which led to a clinical diagnosis of PD.

At the age of 56 years, he developed a wearing-off phenomenon, and the disease was refractory to increasing doses of antiparkinsonian medications. At the age of 57 years, he was admitted to our hospital for a thorough examination and consideration of the indications for device-aided therapy. His daily medication doses were levodopa/benserazide (100 mg), levodopa/carbidopa (450 mg), entacapone (800 mg), rotigotine (22.5 mg), zonisamide (50 mg), selegiline (5 mg), and istradefylline (20 mg). The patient exhibited left-dominant rigidity, a resting tremor, and akinesia/hypokinesia. His gait was highly festinating, shuffling, and slightly wide-based. The finger-nose test revealed mild decomposition and terminal oscillation on the left side; however, ataxia of the lower extremities was not evident. The patient’s Movement Disorder Society Unified Parkinson’s Disease Rating Scale (MDS-UPDRS) part III score was 85 (“off” time) and 50 (“on” time) (41.2% reduction), indicating a clear drug efficacy and wearing-off phenomenon. Constipation, nocturia, and rapid eye movement sleep behavior disorder were also present. His Mini-Mental State Examination total score was 28 out of 30 and his Frontal Assessment Battery score was 13 out of 18, suggesting mild executive dysfunction with no overall cognitive impairment.

Because the patient was a professional truck driver and wished to continue driving, rotigotine was tapered and levodopa was titrated up to 1,000 mg/day. The patient’s MDS-UPDRS part III score was improved from 50 to 31 while on medication. An enteral feeding tube was inserted, and levodopa/carbidopa intestinal gel was introduced. At 1,600 mg/day, the patient’s motor symptoms were under control, but discontinuation of rotigotine induced painful nocturnal leg dystonia.

**Figure 2:**
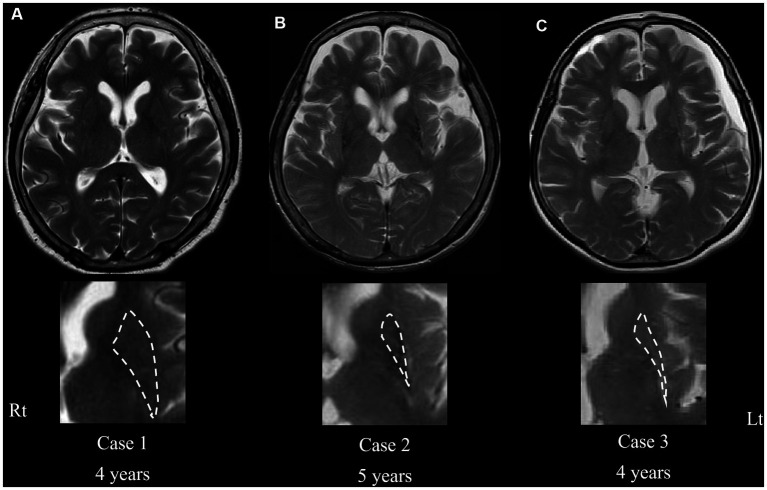
Transaxial T2-weighted magnetic resonance imaging of cases 1 (current case) **(A)**, 2 **(B)**, and 3 **(C)** at the level of the putamen. Severe atrophy is evident in cases 2 and 3 **(B,C)**, whereas no atrophy in the putamen is shown in the current case **(A)**, even in the advanced phase of the disease. Magnified images of the putamen are shown in the bottom column. Dashed lines indicate the border rims of the putamen. The “years” represents the periods from onset.

T2-weighted brain MRI revealed marked cerebellar atrophy, hot cross bun sign, and hyperintensities of the middle cerebellar peduncle (Figure 1C), with no abnormalities in the striatum (Figure 2A). ^123^I-IMP SPECT revealed marked bilateral cerebellar and striatal hypoperfusion (Figure 1D). Autonomic function tests showed no orthostatic hypotension but decreased perspiration and increased post-void urinary residual volume (approximately 300 mL per time). Clinical diagnosis of MSA was established according to Gilman’s diagnostic criteria (9).

Therefore, the device-aided therapy was not recommended. Antiparkinsonian medications, including rotigotine, were resumed at the original doses, which improved the patient’s nocturnal painful leg dystonia.

Approximately a week after discharge, the patient developed active hallucinatory delusions. Gradual reduction in antiparkinsonian medication did not improve his psychiatric symptoms, which led to institutionalization in a nursing home. Later his hallucinations and delusions diminished which led to the cause of the hallucination induced by levodopa. Two weeks later, he experienced cardiopulmonary arrest while defecating in the bathroom and could not be resuscitated.

### Neuropathology of the present case

An autopsy was performed 68 h 52 min after the patient’s death, with the consent of his family. There were no abnormal findings explaining sudden death. A large amount of urine remained in the bladder, and the colon was filled with stool. The brain weighed 1,350 g, with the cerebellum showing modest atrophy, particularly in the white matter (Figure 1E). Furthermore, depigmentation of the SN (Figure 1F) and a slightly brownish putamen with mild atrophy were observed (Figure 1G).

**Figure 3:**
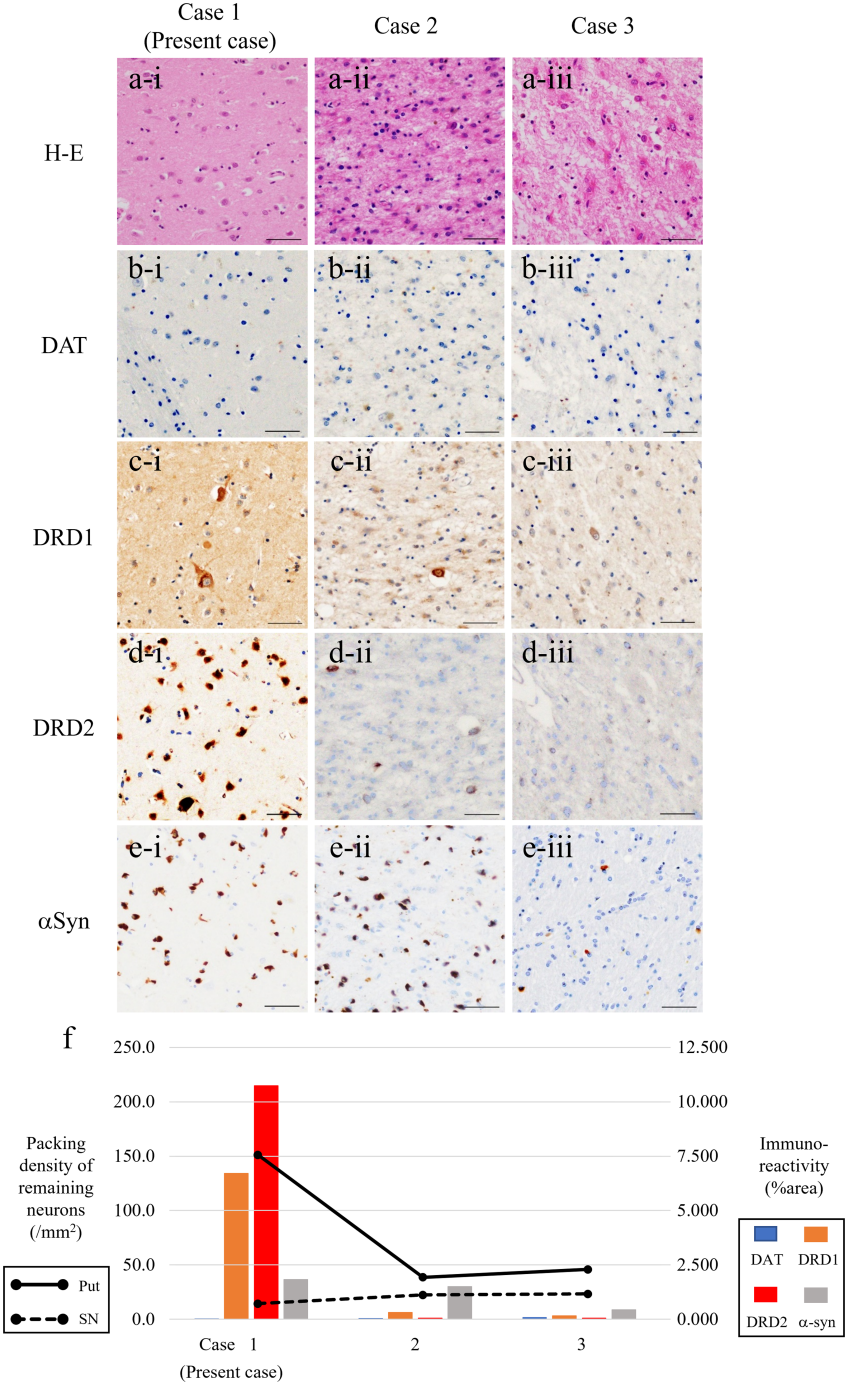
Microphotographs of putamen in three patients with MSA-P. Severe gliosis and neuronal loss are consistent in cases 2 and 3 **(a-ii, iii)** but not in the present case **(a-i)**. Expression of dopamine receptors D1/D2 (DRD1/DRD2) (as postsynaptic markers) are severely reduced in cases 2 and 3 **(c-ii, iii, d-ii, iii)**, but it is well-preserved in the present case **(c-i, d-i)**. Immunoreactivity for dopamine transporter, as a presynaptic marker of nigrostriatal system, is almost reduced in all patients **(b-i–iii)**. Fair amount of α-synuclein (αSyn)-positive GCIs are present in all patients **(e-i–iii)**. **(f)** Line charts represent packing density of remaining neurons in the putamen (solid line) and substantia nigra (dotted line) in patients with MSA-P. Bar charts represent % area of immunoreactivity for DAT (light blue), DRD1 (orange), DRD2 (red), and αSyn (gray columns). Scale bars, 50 μm. Hematoxylin–eosin (H–E) **(a-i–iii)**, DAT **(b-i–iii)**, DRD1 **(c-i–iii)**, DRD2 **(d-i–iii)**, and α-synuclein **(e-i–iii)**. MSA-P, multiple system atrophy with predominant parkinsonism; DAT, dopamine transporter; DRD1, dopamine receptor D1; DRD2, dopamine receptor D2; GCIs, glial cytoplasmic inclusions.

Microscopic examination revealed α-synuclein-positive GCIs, a pathological hallmark of MSA, particularly in the putamen (Figures 3E–I), cerebral white matter, midbrain, pons, medulla oblongata, and cerebellar white matter. Although mild gliosis with large amounts of GCIs was present in the putamen, neuronal loss was not evident or mild, if any was present. Melanin-containing neurons in the locus coeruleus were relatively preserved, whereas those in the SN decreased. Moderate neuronal loss was detected in the pontine nuclei, with pontine transverse fibers showing myelin pallor and abundant GCIs. Moderate gliosis was observed in the cerebellar white matter, whereas the cerebellar cortices were mostly preserved. The inferior olivary nuclei exhibited mild GCIs; however, no neuronal loss was observed. The hippocampus and amygdala were mostly intact. The precentral gyrus was well-preserved, with only a mild presence of GCIs. Although the number of motor neurons was not decreased in the hypoglossal nuclei and spinal anterior horn, only a small number of NCIs were identified. The intermediate lateral nuclei demonstrated evident neuronal loss with a considerable presence of GCIs. The presence of GCIs in the olfactory bulb was extremely mild. Tyrosine hydroxylase-positive cardiac nerve fibers were well-preserved. This finding explained the clinically identified normal uptake of the radioisotope on MIBG scintigraphy. Lewy bodies were not observed, and the minimal amount of tau deposition was limited to the entorhinal cortex (Braak NFT stage 1). No senile plaques were detected in sections stained with Gallyas.

### Comparison between the present patient and other patients with multiple system atrophy with predominant parkinsonism

The demographics and pathological backgrounds of the three patients with MSA-P are presented in Table 1. Although UPDRS scores were not available, two MSA-P patients (cases 2 and 3) poorly responded to levodopa, according to the medical records. The severity of nigral neuronal loss was largely consistent across the entire MSA-P cohort, whereas neuronal loss in the putamen was evident in the other two patients with MSA-P but not in the current patient (Figure 3).

Similarly, DAT immunoreactivity was reduced in all patients, and putaminal expression of both DRD1 and DRD2 severely decreased in the other two patients with MSA but preserved in the present case (Figure 3F). This neuropathological discrepancy indicates that the degeneration of presynaptic nigrostriatal projections was consistent across all patients with MSA-P, whereas the preservation of postsynaptic striatal neurons expressing dopamine receptors was specific to the current patient. Interestingly, there was no putaminal atrophy observed on brain MRI in the present case (Figure 2A); however, atrophy was apparent in the other two patients (cases 2 and 3) undergoing MRI before death (Figures 2B,C). In case 3, there were a small amount of Lewy bodies limited to the dorsal vagal nuclei and locus coeruleus. However, the clinical manifestation in the patient was consistently explained by the presence of large amount of GCIs and neuronal loss in the central nervous system as striatonigral degeneration.

Similar to the findings in the two levodopa-unresponsive patients with MSA-P (cases 2 and 3), the same neuropathological assessment in three patients with pathologically proven MSA-C (one man and two women, pathologically diagnosed from 2007 to 2013) showed severe neuronal loss in the SN and putamen and decreased immunoreactivity of putaminal expression of DAT, DRD1, and DRD2 (Supplementary Table S1, Supplementary Figure S1). These results indicate that pre- and postsynaptic dopaminergic fibers are likely to be vulnerable in patients with MSA, regardless of the clinical subtype, except for the present MSA-P case, which had preserved postsynaptic dopaminergic fibers. Because of the predominant cerebellar ataxia, the three patients with MSA-C were not treated with levodopa throughout the illness period.

## Discussion

This study presented an “atypical” MSA case (the present case) in which levodopa responsiveness was preserved and neuropathologically compared with two levodopa-unresponsive MSA-P cases. In the current case, asymmetrical parkinsonism with good levodopa responsiveness was maintained throughout the 4-year disease course. At the initial visit, imaging findings of brain MRI, DAT scan, and ^123^I-IMP SPECT were consistent with those of PD. Four years after the initial PD diagnosis, increased post-void urinary residual volume, findings in ^123^I-IMP SPECT and MRI, led to a diagnosis of MSA. The final clinical diagnosis was MSA-P; however, imaging and pathological findings showed an olivopontocerebellar (OPC) atrophy pattern, indicating relatively preserved putaminal changes in contrast to nigral impairment. Postmortem study confirmed the diagnosis of MSA, showing relatively preserved neurons in the striatum, as opposed to substantial neuronal loss in the SN. In the present case, the laterality of the parkinsonism (left-dominant) was in accord with the presence of GCIs side in the putamen (right-dominant).

Previous reports have suggested that 30–50% of patients with MSA have good levodopa responsiveness in the early stages of the disease (10–13) and that up to 7% of patients with MSA respond well to levodopa throughout their entire disease course (14). Furthermore, 43 of the 203 (21%) clinically diagnosed patients with MSA are pathologically diagnosed with other neurodegenerative diseases, such as PD or PSP (10). Patients with MSA with little or no striatal degeneration (i.e., preserved postsynaptic dopaminergic systems) usually have good levodopa responsiveness (6, 15), which may account for the degree of degeneration of putaminal neurons expressing dopamine receptors (16, 17). In this postmortem study, when comparing the present patient with other patients, all of them showed a decreased number of neurons in the SN, but there was a marked difference in the density of putaminal neurons and DRD1/DRD2 expression in the putamen. Normal MRI findings of the putamen and decreased striatal DAT binding observed in this patient with levodopa-responsive MSA support the autopsy findings.

In a typical MSA-P, as demonstrated in the other two patients with MSA-P (cases 2 and 3), both presynaptic dopaminergic fibers (i.e., the nigrostriatal pathway) and postsynaptic dopaminergic fibers are impaired, and the possibility of putaminal atrophy on MRI correlating with levodopa responsiveness has been discussed (11). Levodopa responsiveness in MSA patients has been reported to correlate with cell integrity of striatum and substantia nigra, impaired functional coupling of D1 and D2 receptors and changes in nondopaminergic striatal neurotransmission, but the detailed mechanisms remain to be elucidated (7). This study demonstrated a triadic relationship among dopamine receptor preservation, putaminal neuronal preservation, and levodopa responsiveness, providing strong support for the possibility that the degree of putaminal atrophy on MRI may be a predictor of levodopa efficacy. ^11^C-raclopride, a DRD2/DRD3 antagonist radioligand, and PET are capable of evaluating postsynaptic dopaminergic receptor binding, enabling possible discrimination between PD and MSA (18, 19).

Another finding drawn from this case is that the absence of early cerebellar hypoperfusion on ^123^I-IMP SPECT does not exclude the diagnosis of MSA, nor does it rule out the possibility of the later development of cerebellar atrophy. Here, the patient mainly had parkinsonism with good levodopa effectiveness, and the initial ^123^I-IMP SPECT imaging revealed no regional hypoperfusion, including that in the cerebellum. However, patients with PD show relatively increased cerebellar blood flow on SPECT imaging as a compensation for parkinsonism (20). Cerebellar hyperperfusion, which is typically observed in PD, was not evident in this case, possibly indicating a low likelihood of PD. In the current case, it is possible that the cerebellum was already relatively degenerated on the first SPECT scan, thereby avoiding abnormalities in the cerebellar blood flow. Longitudinal neuroimaging observation may provide an answer to the following question: “Where does the degeneration of MSA begin and how does it spread?” Our clinicopathological observations in this case suggested that degeneration initiated in the SN, developed to the OPC regions, and finally reached the putamen. Levodopa-responsive and levodopa-unresponsive MSA may have different underlying pathological spreading processes. Clinically, MSA-P and MSA-C are often distinguished. However, they are mixed and continuous pathologies that are not pathologically independent (21). The autopsy findings in the present case suggested OPC and nigral (presynaptic) pathology, supporting the mixed pathology of both MSA-C and MSA-P.

The main limitation of this study was that we were only able to study a small number of cases, which may not have been sufficient to draw general conclusions. This study was based on a single case of levodopa-responsive MSA-P, and future prospective studies using similar methods are anticipated to contribute to a better understanding of the pathological basis of MSA.

## Conclusion

MSA-P is typically considered levodopa-ineffective because both the pre- and postsynaptic nerves of the dopaminergic pathway are impaired; however, levodopa can be effective if the dopaminergic postsynaptic fibers are preserved. Levodopa efficacy, degree of putaminal volume on MRI, preservation of putaminal neurons, and preservation of DRD1/DRD2 are mutually correlated. Therefore, the degree of putaminal atrophy on MRI is a useful predictor of levodopa efficacy in MSA-P. In the early stages, PD-like clinical phenotypes or imaging findings do not exclude the possibility of MSA, and careful clinical examination and follow-up neuroimaging tests may lead to a more accurate diagnosis.

## Data availability statement

The raw data supporting the conclusions of this article will be made available by the authors, without undue reservation.

## Ethics statement

The studies involving humans were approved by Institutional Review Board of Chiba University. The studies were conducted in accordance with the local legislation and institutional requirements. The participants provided their written informed consent to participate in this study. Written informed consent was obtained from the next of kin for the publication of any potentially identifiable images or data included in this article.

## Author contributions

MT: Investigation, Writing – original draft, Writing – review & editing. TT: Formal analysis, Investigation, Methodology, Validation, Writing – original draft, Writing – review & editing. YK: Investigation, Writing – review & editing. TS: Investigation, Writing – review & editing. KS: Investigation, Writing – review & editing. SH-K: Investigation, Writing – review & editing. TK: Investigation, Writing – review & editing. SK: Supervision, Writing – review & editing. SH: Investigation, Supervision, Writing – original draft, Writing – review & editing.
