# Supplementary material for: Neuropathological features of levodopa-responsive parkinsonism in multiple system atrophy: an autopsy case report and comparative neuropathological study
Source: Front Neurol. 2023 Nov 14;14:1293732. doi: 10.3389/fneur.2023.1293732 (PMC10682068; doi:10.3389/fneur.2023.1293732)
Supplement: Supplementary file 1 [file Data_Sheet_1.DOCX]

**Magnetic resonance imaging (MRI)**

T2 weighted imagings in MRI were obtained using Achieva 3.0 T MR scanner (Phillips, Amsterdam, Netherlands) (thickness: 5 mm; field-of-view: 220 × 220 mm; repetition time: 4297 ms; echo time: 100 ms; flip angle: 90°) in the current patient (case 1), Magnetom Symphony, A Tim System 1.5 T (Siemens, München, Germany) (thickness: 6 mm; field-of-view: 201 × 210 mm; repetition time: 3500 ms; echo time: 86 ms; flip angle: 160°) in the control patients (case 2 and 3).

**^123^I-iodoamphetamine (^123^I-IMP) single-photon emission computed tomography**

15 minutes after the intravenous injection of ^123^I-IMP (dose: 111 MBq), emission data were acquired in a dimly lit room in continuous mode with 3 minutes per cycle over 24-minutes period. This process was facilitated by a dual detector SPECT/CT camera, initial visit: Infinia Hawkeye 4 scanner (General Electric, Boston, USA), four years after onset: NM870DR (General Electric, Boston, USA), both equipped with ELEGP collimators. All emission scans were reconstructed using Butterworth and ramp filters, and attenuation was corrected using Chang's technique (μ = 0.07 cm^−1^). The decreased blood flow was evaluated using three-dimensional stereotactic surface projections (3D-SSP) analysis.

**Metaiodobenzylguanidine (^123^I-MIBG) myocardial scintigraphy**

^123^I-MIBG myocardial scintigraphy was obtained with the intravenous injection of ^123^I-MIBG (dose: 111 MBq), facilitated by Infinia Hawkeye 4 scanner (General Electric, Boston, USA), equipped with ELEGP collimators. 15 minutes after the intravenous injection, emission data were acquired two times, 15 minutes and 3.5 hours post-injection, each 5 minutes. The data were reconstructed by the ordered subset expectation maximization (OSEM) method. Emission scans were reconstructed using Butterworth and ramp filters. Attenuation was corrected using Chang's technique (μ = 0.07 cm^−1^).

**Dopamine transporter (DAT) scan**

DAT scan was obtained with the intravenous injection of ^123^I-ioflupane (^123^I-FP-CIT) (dose: 167 MBq), facilitated by Infinia Hawkeye 4 scanner (General Electric, Boston, USA), equipped with ELEGP collimators. 3 hours after the intravenous injection, emission data were acquired with 3 minutes per cycle over 30-minutes period. The data were reconstructed by the OSEM method. Emission scans were reconstructed using Butterworth and ramp filters. Attenuation was corrected using Chang's technique (μ = 0.07 cm^−1^).
