# Supplementary material for: Neuropathological features of levodopa-responsive parkinsonism in multiple system atrophy: an autopsy case report and comparative neuropathological study
Source: Front Neurol. 2023 Nov 14;14:1293732. doi: 10.3389/fneur.2023.1293732 (PMC10682068; doi:10.3389/fneur.2023.1293732)
Supplement: Supplementary file 2 [file Table_1.DOCX]

Supplementary Table 1: Demographics and neuropathological features of three patients with MSA-C.

| Case | 4 | 5 | 6 |
| --- | --- | --- | --- |
| MSA subtype | MSA-C | MSA-C | MSA-C |
| Age at death (years) | 70 | 59 | 51 |
| Sex | F | M | M |
| Disease duration (years) | 13 | 9 | 3 |
| Putaminal atrophy on MRI | NA | NA | + |
| Levodopa response | NA | NA | NA |
| Brain weight (g) | 900 | 1,280 | 1,430 |
| Lewy pathology | Absent | Absent | Absent |
| Braak NFT staging | 4 | 0 | 0 |

Demographics and neuropathological feature of three cases with MSA-C.

NA: not assessed, NFT: neurofibrillary tangle

Supplementary Figure 1: Microphotographs of putamen in three patients with MSA-C. Severe gliosis and neuronal loss are consistent in all patients (a-iv–vi). Expression of dopamine receptors D1/D2 (DRD1/DRD2) (as postsynaptic markers) are severely reduced in all patients (c-iv–vi, d-iv–vi). Immunoreactivity for dopamine transporter is reduced in all patients (b-iv–vi). Fair amounts of α-synuclein (αSyn)-positive GCIs are present in all patients (e-iv–vi). (f) Line charts represent packing density of remaining neurons in the putamen (solid line) and substantia nigra (dotted line) in patients with MSA-C. Bar charts represent % area of immunoreactivity for DAT (light blue), DRD1 (orange), DRD2 (red), and αSyn (gray columns).

Bars=50 μm

Hematoxylin–eosin (H–E) (a-iv–vi), DAT (b-iv–vi), DRD1 (c-iv–vi), DRD2 (d-iv–vi), and αSyn (e-iv–vi)

MSA-C, multiple system atrophy with a cerebellar variant; DAT, dopamine transporter; DRD1, dopamine receptor D1; DRD2, dopamine receptor D2; GCIs, glial cytoplasmic inclusions
