# Supplementary figures and images for: Neuropathological features of levodopa-responsive parkinsonism in multiple system atrophy: an autopsy case report and comparative neuropathological study
Source: Front Neurol. 2023 Nov 14;14:1293732. doi: 10.3389/fneur.2023.1293732 (PMC10682068; doi:10.3389/fneur.2023.1293732)

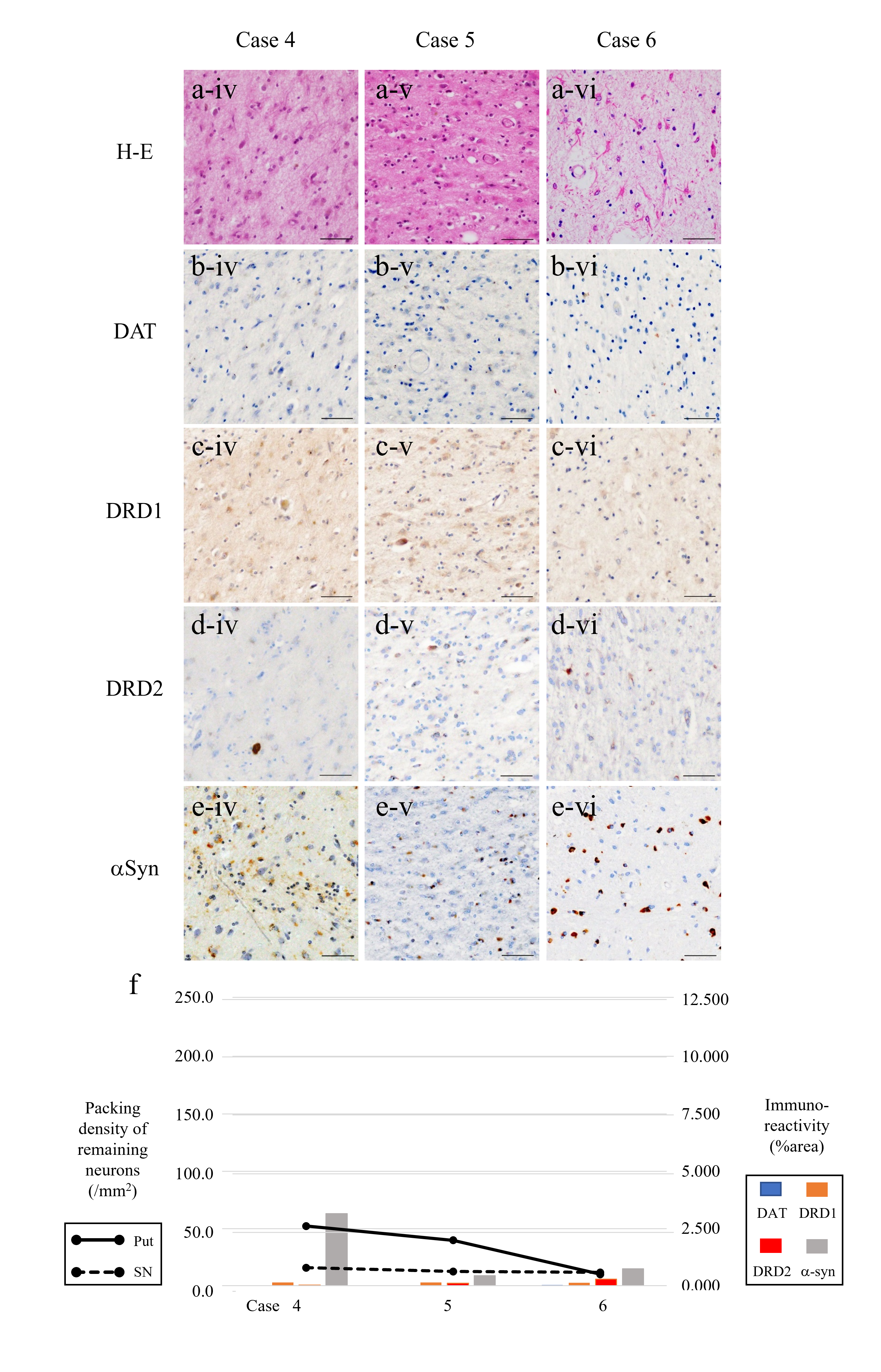

Supplement: Supplementary file 3 [file Image_1.tif]
